# Investigation of the fine structure of antihydrogen

**DOI:** 10.1038/s41586-020-2006-5

**Published:** 2020-02-19

**Authors:** The ALPHA Collaboration, M. Ahmadi, B. X. R. Alves, C. J. Baker, W. Bertsche, A. Capra, C. Carruth, C. L. Cesar, M. Charlton, S. Cohen, R. Collister, S. Eriksson, A. Evans, N. Evetts, J. Fajans, T. Friesen, M. C. Fujiwara, D. R. Gill, P. Granum, J. S. Hangst, W. N. Hardy, M. E. Hayden, E. D. Hunter, C. A. Isaac, M. A. Johnson, J. M. Jones, S. A. Jones, S. Jonsell, A. Khramov, P. Knapp, L. Kurchaninov, N. Madsen, D. Maxwell, J. T. K. McKenna, S. Menary, J. M. Michan, T. Momose, J. J. Munich, K. Olchanski, A. Olin, P. Pusa, C. Ø. Rasmussen, F. Robicheaux, R. L. Sacramento, M. Sameed, E. Sarid, D. M. Silveira, C. So, D. M. Starko, G. Stutter, T. D. Tharp, R. I. Thompson, D. P. van der Werf, J. S. Wurtele

**Affiliations:** 1 Department of Physics, University of Liverpool, Liverpool, UK; 2 Department of Physics and Astronomy, Aarhus University, Aarhus, Denmark; 3 Department of Physics, College of Science, Swansea University, Swansea, UK; 4 School of Physics and Astronomy, University of Manchester, Manchester, UK; 5 Cockcroft Institute, Warrington, UK; 6 TRIUMF, Vancouver, British Columbia Canada; 7 Department of Physics, University of California at Berkeley, Berkeley, CA USA; 8 Instituto de Fisica, Universidade Federal do Rio de Janeiro, Rio de Janeiro, Brazil; 9 Department of Physics, Ben-Gurion University of the Negev, Beer-Sheva, Israel; 10 Department of Physics and Astronomy, University of Calgary, Calgary, Alberta Canada; 11 Department of Physics and Astronomy, University of British Columbia, Vancouver, British Columbia Canada; 12 Department of Physics, Simon Fraser University, Burnaby, British Columbia Canada; 13 Department of Physics, Stockholm University, Stockholm, Sweden; 14 Department of Physics and Astronomy, York University, Toronto, Ontario Canada; 15 École Polytechnique Fédérale de Lausanne (EPFL), Swiss Plasma Center (SPC), Lausanne, Switzerland; 16 Department of Chemistry, University of British Columbia, Vancouver, British Columbia Canada; 17 Department of Physics and Astronomy, University of Victoria, Victoria, British Columbia Canada; 18 Department of Physics and Astronomy, Purdue University, West Lafayette, IN USA; 19 Soreq NRC, Yavne, Israel; 20 Physics Department, Marquette University, Milwaukee, WI USA; 21 IRFU, CEA/Saclay, Gif-sur-Yvette, France

**Keywords:** Exotic atoms and molecules, Experimental particle physics

## Abstract

At the historic Shelter Island Conference on the Foundations of Quantum Mechanics in 1947, Willis Lamb reported an unexpected feature in the fine structure of atomic hydrogen: a separation of the 2S_1/2_ and 2P_1/2_ states^1^. The observation of this separation, now known as the Lamb shift, marked an important event in the evolution of modern physics, inspiring others to develop the theory of quantum electrodynamics^2–5^. Quantum electrodynamics also describes antimatter, but it has only recently become possible to synthesize and trap atomic antimatter to probe its structure. Mirroring the historical development of quantum atomic physics in the twentieth century, modern measurements on anti-atoms represent a unique approach for testing quantum electrodynamics and the foundational symmetries of the standard model. Here we report measurements of the fine structure in the *n* = 2 states of antihydrogen, the antimatter counterpart of the hydrogen atom. Using optical excitation of the 1S–2P Lyman-α transitions in antihydrogen^6^, we determine their frequencies in a magnetic field of 1 tesla to a precision of 16 parts per billion. Assuming the standard Zeeman and hyperfine interactions, we infer the zero-field fine-structure splitting (2P_1/2_–2P_3/2_) in antihydrogen. The resulting value is consistent with the predictions of quantum electrodynamics to a precision of 2 per cent. Using our previously measured value of the 1S–2S transition frequency^6,7^, we find that the classic Lamb shift in antihydrogen (2S_1/2_–2P_1/2_ splitting at zero field) is consistent with theory at a level of 11 per cent. Our observations represent an important step towards precision measurements of the fine structure and the Lamb shift in the antihydrogen spectrum as tests of the charge–parity–time symmetry^8^ and towards the determination of other fundamental quantities, such as the antiproton charge radius^9,10^, in this antimatter system.

## Abstract (web summary)

Precision measurements of the 1S–2P transition in antihydrogen that take into account the standard Zeeman and hyperfine effects confirm the predictions of quantum electrodynamics.

## Main

The fine-structure splitting of the *n* = 2 states of hydrogen is the separation of the 2P_3/2_ and 2P_1/2_ levels at zero magnetic field. This splitting, predicted by the Dirac theory of relativistic quantum mechanics^11^, originates from the spin–orbit interaction between the non-zero orbital angular momentum (*L* = 1) and the electron spin. The ‘classic’ Lamb shift is defined as the splitting between the 2S_1/2_ and 2P_1/2_ states at zero field^12^, and is a manifestation of the interaction of the electron with the quantum fluctuations of the vacuum electromagnetic field, an effect explained by quantum electrodynamics (QED)^12–14^. Today, it is understood that the classic Lamb shift in hydrogen is dominated by the QED effects on the 2S energy level, and that the 1S level receives even stronger QED corrections than the 2S level^12,13^. Although QED corrections in levels *n* ≠ 2 are now also sometimes referred to as Lamb shifts, in this Article we restrict our definition of the Lamb shift to be the classic *n* = 2 shift.

**Fig. 1:**
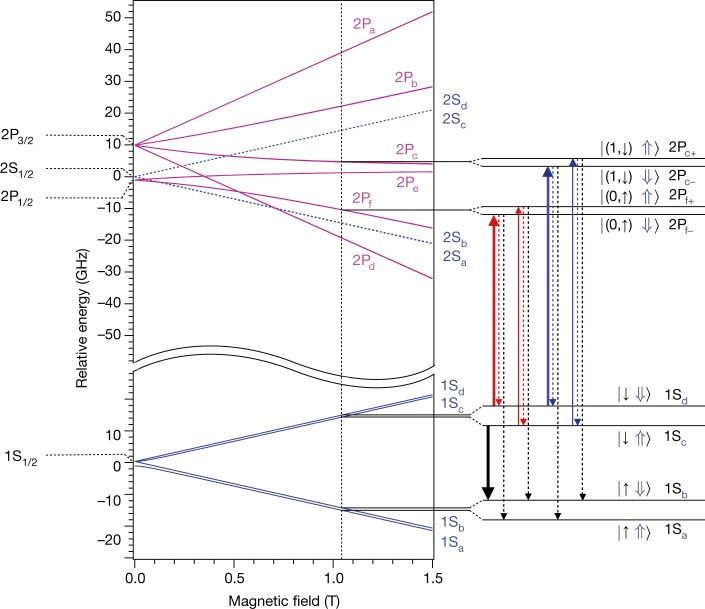
Expected antihydrogen energy levels. Calculated energies of the fine structure and the hyperfine sublevels of the 1S_1/2_, 2S_1/2_, 2P_3/2_ and 2P_1/2_ states are shown as functions of magnetic-field strength. The spin orientations for antihydrogen are shown; they are reversed for hydrogen. The centroid energy difference, *E*_1S–2S_ = 2.4661 × 10^15^ Hz, has been suppressed on the vertical axis. Details of the energy levels relevant to this work at a magnetic field of *B* = 1.0329 T are shown on the right. Each state is labelled using conventional notation. For the 1S and 2S states, the hyperfine states are labelled with subscripts a–d in order of increasing energy (see, for example, ref. ^7^); namely, ${{\rm{S}}}_{{\rm{a}}}=|\uparrow \Uparrow \rangle$, ${{\rm{S}}}_{{\rm{b}}}=|\uparrow \Downarrow \rangle$, ${{\rm{S}}}_{{\rm{c}}}=|\downarrow \Uparrow \rangle$ and ${{\rm{S}}}_{{\rm{d}}}=|\downarrow \Downarrow \rangle$, where the ket notation represents the positron spin (left; ↓ or ↑) and antiproton spin (right; ⇓ or ⇑) states in the high-field limit. The labels S_ab_ and S_cd_ are used when the antiproton spins are unpolarized. For the 2P states, the fine-structure splittings are labelled with subscripts a–f in order of decreasing energy at low magnetic fields, whereas the hyperfine splitting due to the antiproton spin is specified by subscripts + and − for spin parallel (⇑) and anti-parallel (⇓) to the magnetic field in the high-field limit, respectively. The symbol (↓,↑) in the figure indicates that the positron spin states are mixed for the 2P_c_ and 2P_f_ states. The vertical solid arrows indicate the one-photon laser transitions probed here: 1S_d_ → 2P_f−_ (bold red), 1S_c_ → 2P_f+_ (thin red), 1S_d_ → 2P_c−_ (bold blue) and 1S_c_ → 2P_c+_ (thin blue). The dashed red and blue arrows indicate relaxation to the same trappable level, which is not detectable in the present experiment, and the dashed black arrows indicate relaxation to untrappable levels, which is detectable via annihilation signals (see text). The bold black arrow shows the microwave transition used to eliminate 1S_c_ state atoms to prepare a doubly spin-polarized antihydrogen sample.

In a magnetic field, the Zeeman effect causes the 2P_3/2_ state to also split into four sublevels (labelled 2P_a_, 2P_b_, 2P_c_ and 2P_d_), whereas the 2S_1/2_ and 2P_1/2_ states each split into two (2S_ab_ and 2S_cd_; 2P_e_ and 2P_f_). These fine-structure levels further split into two hyperfine states owing to the proton spin (see Fig. 1 for the expected energy levels for the case of antihydrogen, where the spin orientations are reversed with respect to those of hydrogen.)

Lamb’s original work used the then newly developed techniques of an excited-state atomic hydrogen beam and resonant microwave spectroscopy to study direct transitions between the *n* = 2 fine-structure states in various magnetic fields. The Lamb shift was then determined to 10% precision by extrapolating frequency measurements to zero field^1^. Here, we report the observation of the splitting between the 2P_c_ and 2P_f_ states in antihydrogen in a field of 1 T, by studying laser-induced transitions from the ground state. Assuming the validity of the Zeeman and hyperfine interactions, and using the value of the previously measured 1S–2S transition frequency^7^, we infer from our results the values of the zero-field fine-structure splitting and the classic Lamb shift in antihydrogen. Such studies have become possible owing to the combination of several recent advances: the accumulation^15^ of hundreds of anti-atoms in each run, their confinement for many hours^16^, control of the hyperfine polarization of the antihydrogen samples^17^ and the development of a narrow-line, pulsed, Lyman-α laser^6,18^.

**Fig. 2:**
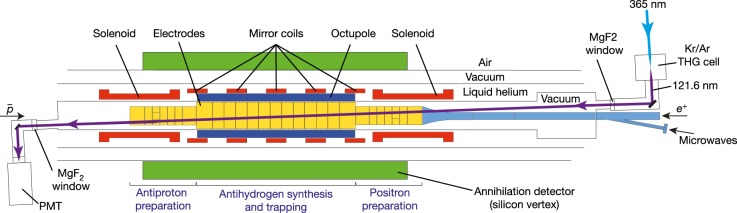
The ALPHA-2 central apparatus. A cylindrical trapping volume for neutral antimatter with a diameter of 44.35 mm and an axial length of 280 mm is located inside several Penning trap electrodes and surrounded by an octupole coil, five mirror coils and two solenoids, all superconducting. The three-layer silicon vertex annihilation detector is shown schematically in green. Laser light (purple line) enters from the positron (*e*^+^) side (right) and is transmitted to the antiproton ($\bar{p}$) side (left) through vacuum-ultraviolet-grade MgF_2_ ultrahigh-vacuum windows. The laser beam crosses the trap axis at an angle of 2.3°. The transmitted 121.6-nm pulses are detected by a solar-blind photomultiplier tube (PMT) at the antiproton side. Microwaves used to prepare the doubly spin-polarized samples are introduced from the positron side through a waveguide, shown in blue. The external solenoid magnet for the Penning traps is not shown here. THG, third-harmonic generation.

Details of the production, trapping and control of antihydrogen in the ALPHA experiment have been provided elsewhere^6,7,15–25^, so the following description is brief. The ALPHA-2 apparatus (Fig. 2) incorporates a cylindrical magnetic trapping volume (about 400 cm^3^) for neutral anti-atoms; the magnetic-field minimum at the centre of the trap was set to 1.0329 ± 0.0004 T for this work. (All uncertainties given herein are 1*σ*.) By combining 90,000 trapped antiprotons from the CERN Antiproton Decelerator^23^ and three million positrons from a positron accumulator^24,25^, about 10–30 cold (below 0.54 K) anti-atoms are confined in the magnetic trap in a 4-min cycle. Under normal conditions, the storage lifetime^16^ of the trapped antihydrogen is greater than 60 h, which permits loading from repeated cycles^15^ to obtain hundreds of antihydrogen atoms in a few hours.

Two types of antihydrogen samples were used in these studies. The positron spin of an antihydrogen atom confined in the ALPHA-2 trap is necessarily polarized, because only the 1S_c_ and 1S_d_ states can be magnetically trapped (Fig. 1). The antiproton spin, on the other hand, is unpolarized a priori, with both orientations equally likely. Thus, the initial samples are singly spin-polarized. On the other hand, doubly spin-polarized samples, in which both the positron and antiproton spins are polarized, can be prepared by injecting microwaves to resonantly drive the 1S_c_ atoms to the untrappable 1S_b_ state (Fig. 1), effectively depopulating the 1S_c_ state from the trap^17^.

Spectroscopy in the vacuum ultraviolet range is challenging even for ordinary atoms, owing in part to the lack of convenient laser sources and optical components^26–28^. Our pulsed, coherent 121.6-nm radiation was produced by generating the third harmonic of 365-nm pulses in a Kr/Ar gas mixture at a repetition rate of 10 Hz (ref. ^18^). The typical pulse width at 121.6 nm was 12 ns, and the bandwidth was estimated from the Fourier transform of the temporal pulse shape to be 65 MHz (full-width at half-maximum, FWHM). The 121.6-nm light was linearly polarized because of the three-photon mixing of linearly polarized 365-nm light. In the antihydrogen trap, the polarization vector was nearly perpendicular to the direction of the axial magnetic field. The laser beam had a radius of 3.6 mm and was roughly collimated across the trapping region (Fig. 2). The average pulse energies in the antihydrogen trapping volume ranged from 0.44 nJ to 0.72 nJ over different runs, as evaluated from the pulse waveforms recorded with a calibrated, solar-blind photomultiplier detector.

In this experiment, single-photon transitions from the 1S_c_ (1S_d_) states to the 2P_c+_ (2P_c−_) and 2P_f+_ (2P_f−_) states are driven by the 121.6-nm light (red and blue arrows in Fig. 1). When antihydrogen is excited to the 2P_c±_ or 2P_f±_ state, it decays to the ground-state manifold within a few nanoseconds by emitting a photon at 121.6 nm. The mixed nature of the positron spin states in the 2P_c+_ (2P_c−_) and 2P_f+_ (2P_f−_) states implies that these states can decay to the 1S_b_ (1S_a_) states via a positron spin flip (black dashed arrows in Fig. 1). Atoms in these final states are expelled from the trap and are annihilated on the trap walls. Annihilation products (charged pions) are in turn detected by a silicon vertex detector^29^ with an efficiency greater than 80%.

Table 1 summarizes our data. In total, four series of measurements were performed using either singly or doubly spin-polarized samples. The Series 1 data, previously reported in ref. ^6^, have been reanalysed. Each series consisted of two or four runs, and in each run about 500 antihydrogen atoms were accumulated over approximately two hours, typically involving over 30 production cycles. The trapped anti-atoms were then irradiated for about two hours by a total of 72,000 laser pulses at twelve different frequencies (that is, 6,000 pulses per frequency point for each run) spanning the range −3.10 GHz to +2.12 GHz relative to the expected (hydrogen) transition frequencies. The laser frequency was changed every 20 s in a non-monotonic fashion to minimize effects related to the depletion of the sample of antihydrogen. After the laser exposure, the remaining antihydrogen atoms were released by shutting down the trap magnets, typically in 15 s, and counted via detection of their annihilation events. 40–60% of the trapped antihydrogen atoms experienced resonant, laser-induced spin flips, and their annihilations were detected during the two-hour laser irradiation period.

**Table 1:** Experimental parameters and number of detected events

Series	Sample polarization	Transition probed	Number of runs	Average pulse energy (pJ)	Number of frequencies	Number of pulses per frequency	Number of trapped atoms	Microwave counts	Laser counts	Counts upon release
1	Single	1S_cd_→2P_c±_	4	600	12	24,000	2,004	–	1,197	807
2	Single	1S_cd_→2P_f±_	4	550	12	24,000	2,012	–	1,075	937
3	Double	1S_d_→2P_c−_	2	440	12	12,000	1,044	527	229	288
4	Double	1S_d_→2P_f−_	2	720	12	12,000	971	463	341	167

The experimental parameters, together with the number of antihydrogen events detected during the microwave irradiation, the laser irradiation and the release of the remaining atoms, are tabulated for each series. The machine-learning analysis identifies annihilation events with an estimated efficiency of 0.849 for the microwave irradiation, 0.807 for the laser irradiation and 0.851 for the release of the remaining atoms. The number of counts is corrected for the detection efficiencies. The number of trapped atoms is derived from the sum of the other counts.

A combination of time-gated antihydrogen detection (enabled by the use of a pulsed laser), the accumulation of a large number of anti-atoms and the use of supervised machine-learning analysis^29^ (based on a boosted decision-tree classifier) suppressed the background to a negligible level (less than 2 counts per 2-h irradiation period).

**Fig. 3:**
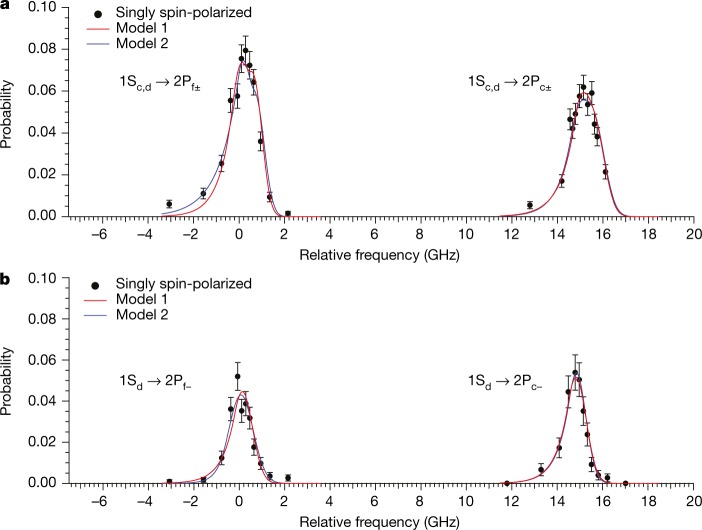
1S–2P fine-structure spectrum of antihydrogen. **a**, **b**, Experimental data (filled circles) and fitted lineshapes for singly spin-polarized (**a**) and doubly spin-polarized (**b**) antihydrogen samples. The data points were obtained from the detected spin-flip events, normalized to the total number of trapped antihydrogen atoms, for a laser pulse energy of 0.5 nJ. The error bars are 1*σ* counting uncertainties. The frequency is offset by 2,466,036.3 GHz. We note that no data were taken between the two peaks (~2–12 GHz). The red fit curves were obtained via our standard fitting procedure (Model 1), and the blue curves were derived from an alternative fitting model (Model 2), illustrating the sensitivity of our results to the fitting procedure. See text and Methods for detailed discussion.

The measured spectra, obtained from counting the laser-induced spin-flip events, are shown in Fig. 3 for both singly and doubly spin-polarized antihydrogen samples. For each run, the probability at each frequency point is determined from dividing the number of annihilation events recorded at that frequency by the total number of trapped atoms in that run, and further dividing by the ratio of the average laser energy to a standard value of 0.5 nJ. The normalization to the standard laser energy is to account for the expected linear dependence of the transition probability on the laser power in our regime. The data plotted in Fig. 3 are spectrum-averaged over the runs for each series. For the singly polarized sample (Fig. 3a), each transition shows a linewidth of about 1.5 GHz (FWHM). This is consistent with the expected Doppler broadening in our trapping condition (1 GHz FWHM) and the hyperfine splitting of the 1S–2P_f_ and 1S–2P_c_ transitions (0.71 GHz for both transitions). The hyperfine structure cannot be resolved in these singly polarized samples owing to the Doppler broadening.

Figure 3b shows the spectra obtained from doubly spin-polarized antihydrogen samples. For these data, microwave radiation of ~28 GHz (power ~0.4 W, measured at the trap entrance) was applied before the start of optical spectroscopy, in the form of a 9-MHz sweep, covering the 1S_c_–1S_b_ transition in the magnetic-field minimum^17^. As shown in Table 1, about half of the total trapped antihydrogen atoms underwent a positron spin-flip and annihilated during microwave irradiation. This is consistent with our experience from earlier studies, in which 1S_c_-state atoms were removed with about 95% efficiency^7,17^. The spectral lines of the 1S–2P transitions in doubly spin-polarized antihydrogen (Fig. 3b) are narrower than those in the singly spin-polarized samples (Fig. 3a) because the former involves only one hyperfine state in the ground state. The peaks are red-shifted because the frequencies of the transition from the 1S_d_ state to the 2P_f_ and 2P_c_ states are expected to be about 700 MHz lower than those from the 1S_c_ state. The observed width of ~1 GHz FWHM of these lines is in agreement with the Doppler width expected for our trapping conditions.

**Extended Data Fig. 1:**
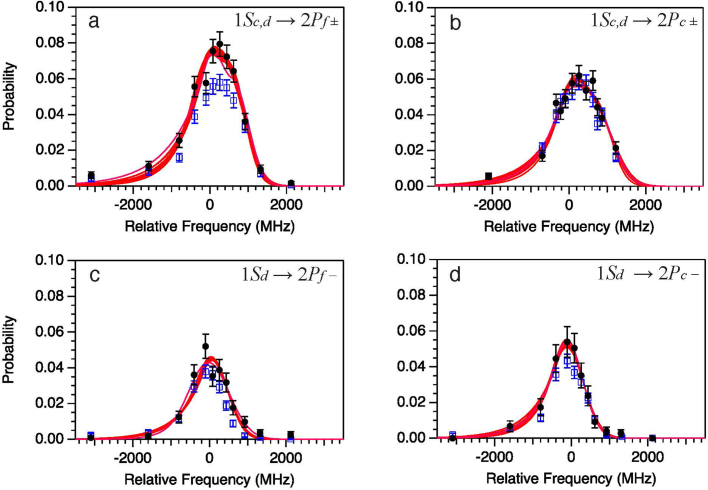
Determination of transition frequencies. **a**–**d**, For each series, the experimental data (filled black circles with error bars) are plotted with fits of various models (red lines) discussed in Methods. The experimental data are normalized to the total number of the detected antihydrogen atoms and a laser power of 5 nW. Also shown are the results of standard simulations (open blue squares with error bars), similarly normalized to the total number of simulated atoms, illustrating the degree of agreement between the data and the simulations, without any tuning parameters. Some discrepancies in the amplitudes can be observed, which may point to errors in our laser power estimates. We note that because our frequency-fitting procedure allows variations in the relative amplitudes, the fits are largely insensitive to the amplitude differences (Methods). Error bars represent 1*σ*.

The procedure used to extract the frequencies of the fine-structure transitions and to evaluate their associated uncertainties is described in Methods. We summarize the results of this analysis in Table 2. A simulation was used to model the motion of trapped antihydrogen atoms in the ALPHA-2 trap and their interaction with pulsed laser radiation. The resonance transition frequencies were obtained by comparing simulated and experimental lineshapes. Extensive investigations were performed to evaluate systematic uncertainties in our measurement (Table 3). The validity of our analysis procedure was tested by using different lineshape-fitting models. Two representative curve fits are shown in Fig. 3. The fit of Model 1 uses a function constrained to fit the simulation shape, whereas in Model 2 the shape parameters of this function are allowed to vary to best fit the experimental data; see Methods for details. The sensitivity of the results to the experimental and simulation parameters was tested by repeating the analysis procedure for a number of simulations with varied input. These included the initial antihydrogen conditions (such as the initial temperature, the quantum state, and the cloud diameter of antihydrogen at formation) and laser properties (such as linewidth, beam waist size and beam position); see Methods and Extended Data Fig. 1. Other sources of systematic uncertainties include the calibration accuracy and a possible frequency drift of the wavemeter, frequency shifts of the 730-nm amplification laser cavity, and possible incomplete clearing of the 1S_c_ state in the preparation of the doubly spin-polarized samples (Table 3 and Methods).

**Table 2:** 1S–2P transition frequencies

	Sample spin polarization	Antihydrogen *f*_res_(exp) (MHz)	Hydrogen *f*_res_(th) (MHz)	Difference *f*_res_(exp) − *f*_res_(th) (MHz)
1S_cd_→2P_c±_	Single	2,466,051,659(62)	2,466,051,625	34
1S_cd_→2P_f±_	Single	2,466,036,611(88)	2,466,036,642	−31
1S_d_→2P_c−_	Double	2,466,051,189(76)	2,466,051,270	−81
1S_d_→2P_f−_	Double	2,466,036,395(81)	2,466,036,287	108

The experimentally determined transition frequencies for antihydrogen *f*_res_(exp) (with 1*σ* errors in parentheses) are compared with the theoretically expected values for hydrogen *f*_res_(th) at a magnetic field of 1.0329 T. For the singly spin-polarized data, the centroid of the hyperfine states is given. The transition frequencies for hydrogen were calculated to a precision better than 1 MHz (Methods).

**Table 3:** Summary of uncertainties

Source of uncertainty	1S_d_→2P_c−_ Doubly spin-polarized (MHz)	1S_d_→2P_f−_ Doubly spin-polarized (MHz)	1S_cd_→2P_c±_ Singly spin-polarized (MHz)	1S_cd_→2P_f±_ Singly spin-polarized (MHz)
Lineshape fit statistics	55	54	45	47
Fitting-model dependence	24	42	17	62
Wavemeter drift	30	30	30	30
Wavemeter offset	18	18	18	18
730-nm cavity frequency correction	18	18	18	18
Residual 1S_c_ state atoms in doubly spin-polarized sample	23	16	0	0
Magnetic field	5	8	5	8
Total	76	81	62	88

Estimated uncertainties (1*σ*) at 121.6 nm for each transition (Methods).

**Fig. 4:**
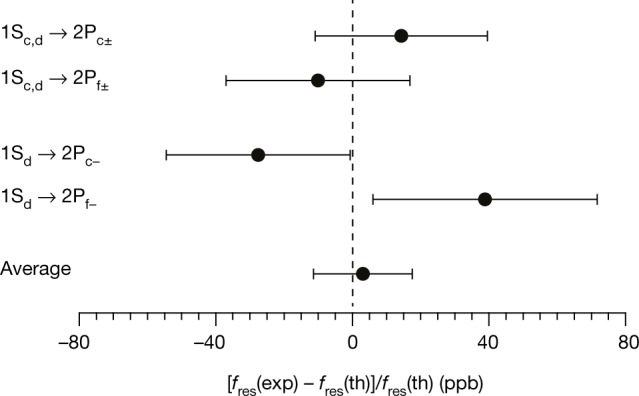
Comparison of antihydrogen and hydrogen transition frequencies. The experimentally measured frequencies for the 1S–2P transitions in antihydrogen *f*_res_(exp) are compared with those theoretically expected for hydrogen *f*_res_(th) (Table 2). All four measurements are consistent with hydrogen, and their average gives a combined test of CPT invariance at 16 parts per billion (ppb). The error bars are 1*σ*, and the calculation of the error bar for the average takes into account correlated uncertainties (Methods).

Within the uncertainties, the measured transition frequencies agree with theoretical expectations for hydrogen for all four series (Table 2, Fig. 4). The fact that the four measurements are consistent, despite having different systematics, increases the confidence in our overall results. The results can be combined to give a test of charge–parity–time (CPT) invariance in the 1S–2P transitions at the level of 16 parts per billion (Fig. 4).

Fundamental physical quantities of antihydrogen can be extracted from our optical measurements of the 1S–2P transitions by combining them with our earlier measurement of the 1S–2S transition in the same magnetic trapping field^7^. From the weighted average of the results between the singly polarized and doubly polarized measurements (Table 1), we obtain a 2P_c−_–2P_f−_ splitting of 14.945 ± 0.075 GHz, a 2S_d_–2P_c−_ splitting of 9.832 ± 0.049 GHz and a 2S_d_–2P_f−_ splitting of 24.778 ± 0.060 GHz at 1.0329 T (Methods). Only two of these three splittings are independent, and they all agree with the values predicted for hydrogen in the same field.

In interpreting our data, we categorize features in the spectrum based on the order of the fine-structure constant *α* in a perturbative series expansion in quantum field theory (which is assumed to be valid for the purpose of our categorization). Those features that can be described by the Dirac theory (the Zeeman, hyperfine and fine-structure effects) are referred to as ‘tree-level effects’ and follow from the lower-order terms (up to order ~*α*^2^Ry, where Ry is the Rydberg constant). On the other hand, the Lamb shift originates from the so-called ‘loop effects’ (order ~*α*^3^Ry*)*, the calculation of which requires the concept of renormalization to avoid infinities^12–14^. Each of the measured splittings has different sensitivity to different terms. At the level of our precision, the 2P_c_–2P_f_ splitting is sensitive to the tree-level terms with negligible QED effects, whereas the 2S–2P_f_ and 2S–2P_c_ splittings are sensitive to the field-independent Lamb shift, in addition to the tree-level terms (we note that the Lamb shift is predicted to have negligible dependence on the magnetic field^14^). The agreement between our measurement and the Dirac prediction for the 2P_c−_–2P_f−_ splitting supports the consistency of the tree-level theory in describing the Zeeman, hyperfine and fine-structure interactions in the 2P states of antihydrogen. If we hence assume that we can correctly account for the tree-level effects in our measurements, we can infer from our measured splittings the values of the zero-field fine-structure splitting in antihydrogen to be 10.88 ± 0.19 GHz. By combining the current result with the much more precisely measured 1S–2S transition frequency in antihydrogen^7^, we obtain a classic Lamb shift of 0.99 ± 0.11 GHz (Methods). If we use the theoretical value of the fine-structure splitting from the Dirac prediction (rather than treat it as a parameter), we can derive a tighter constraint on the Lamb shift, 1.046 ± 0.035 GHz.

When considering the first measurements on an exotic system such as antihydrogen, it is necessary to adopt a framework within which it is possible to compare the results to the expectations of well established models for normal matter. The choice of which effects can be assumed to be true in interpreting the data are, of necessity, somewhat arbitrary. The approach illustrated here is based on the order of perturbation in the coupling constant *α*; we have assumed (lower-order) tree-level effects in order to extract (higher-order) renormalizable loop effects. Other approaches are possible in interpreting our data. We note that if the standard theory for the hydrogen atom applies to antihydrogen, most of the expected QED effect is on the 2S level, rather than on the 2P level. Furthermore, the 1S level receives approximately *n*^3^ = 8 times larger QED corrections than the 2S level; hence, our earlier accurate determination of the antihydrogen 1S–2S level difference^7^ gives strong constraints on new interactions within the QED framework. However, it is possible that a new effect could show up in the antihydrogen classic Lamb shift while satisfying the 1S–2S constraint. See ref. ^8^ for an example in a Lorentz-violating effective-field theory framework.

We have investigated the fine structure of the antihydrogen atom in the *n* = 2 states. The splitting between the 2P_c_ and 2P_f_ states, two of the 2P Zeeman sublevels belonging to the *J* = 3/2 and *J* = 1/2 manifolds (*J*, total angular momentum), has been observed in a magnetic field of 1 T. The energy levels of the 1S–2P transitions agree with the Dirac theory predictions for hydrogen at 1 T to 16 parts per billion, and their difference to 0.5%. By assuming the standard Zeeman and hyperfine effects, and by combining our results with the earlier result of 1S–2S spectroscopy^7^, we have inferred the zero-field fine-structure splitting and the classic Lamb shift in the *n* = 2 level.

These observations expand the horizons of antihydrogen studies, providing opportunities for precision measurements of the fine structure and the Lamb shift—both of which are longstanding goals in the field. Prospects exist for considerable improvements in the precision beyond this initial determination. With the advent of the ELENA ring in 2021, an upgrade to the Antiproton Decelerator with an anticipated increase in the antiproton flux, the statistical uncertainties are expected to be dramatically reduced. The development of laser cooling^30^ would reduce the Doppler width to a level comparable to the natural linewidth, which in turn would improve the precision of the frequency determination. It would also permit direct experimental determination of the hyperfine splitting in the 2P states, for which theoretical values were assumed in this study.

Such measurements will provide tests of CPT invariance that are complementary to other precision measurements in antihydrogen, such as the 1S–2S frequency and the ground-state hyperfine splitting. Furthermore, a precise value of the classic Lamb shift, combined with that of the 1S–2S interval, will permit an antimatter-only determination of the antiproton charge radius^9,10^, without referring to matter measurements—that is, independent of the proton charge radius puzzle^31–33^. These examples signify the importance of broad and complementary measurements in testing fundamental symmetries. In the absence of compelling theoretical arguments to guide the way to possible asymmetries, it is essential to address the antihydrogen spectrum as comprehensively as is practical. Finally, the results reported here demonstrate our capability to precisely and reproducibly drive vacuum ultraviolet transitions on a few anti-atoms, and indicate our readiness for laser cooling of antihydrogen^30^, an eagerly anticipated development in antimatter studies with far-reaching implications for both spectroscopic and gravitational studies^34^.

## Methods

### Transition-frequency determination

The observed 1S–2P transition spectra have asymmetric shapes with a low-frequency tail caused by Zeeman shifts in the inhomogeneous-magnetic-field regions away from the centre of the ALPHA-2 trap. As a result, the apparent peak of the observed spectrum is shifted to a slightly lower frequency with respect to the resonance transition frequency *f*_res_, which is defined for atoms in resonance at the magnetic-field minimum of the trap. This offset is relatively small (of the order of 50 MHz). Nonetheless, we performed extensive analysis to understand the effects of this asymmetry on our transition-frequency determination. The details of the analysis follow.

A detailed simulation was used to model the motion of trapped antihydrogen atoms in the ALPHA-2 trap, as well as their interaction with pulsed laser radiation. Aspects of our simulation have been validated in previous studies (for example, refs. ^10,11,19–24^). To determine the resonance transition frequency, we first simulated lineshapes for the transitions from the two trappable 1S hyperfine states to the 2P_c_ and 2P_f_ excited states (that is, for four transitions: 1S_c_ → 2P_c+_, 1S_c_ → 2P_f+_, 1S_d_ → 2P_c−_ and 1S_d_ → 2P_f−_). We then fitted each component with an asymmetric lineshape function, referred to as GE. GE is a Gaussian spliced to an exponential low frequency tail, where the derivative of the crossover point is required to be continuous. GE has four parameters: the peak frequency (*f*_peak_) and the width (*W*) of the Gaussian, the crossover point frequency (*f*_x_) and the overall amplitude (*A*). From the fit, we determined the simulated lineshape parameters *f*_peak_(sim), *W*(sim), *f*_x_ (sim) and *A*(sim) for each transition. In addition, we derived the peak frequency offset Δ*f*, defined as Δ*f* = *f*_peak_(sim) − *f*_res_(th), where *f*_res_(th) is the expected theoretical resonance frequency for hydrogen in the magnetic field *B*.

The experimentally observed spectra were then fitted with GE lineshapes. A sum of two GEs was used to fit singly spin-polarized samples, where only *f*_peak_ and a single normalization factor were used as the fitting parameters, whereas the rest of the parameters (that is, the *W* and *f*_x_ values of each GE, the spacing of *f*_peak_ between two GEs, and the ratio of the amplitudes *A* of two GEs) were fixed to the corresponding simulated values. For doubly spin-polarized samples, the experimental spectra were fitted with a single GE lineshape. In these fits, *W* and *f*_x_ were fixed using a fit to the simulated spectrum in which an estimated 5% contamination of the 1S_c_ component was assumed. The experimental transition frequency is given by *f*_res_(exp) = *f*_peak_(exp) − Δ*f*, where *f*_peak_(exp) is the peak frequency of the experimental data obtained by the fit. Here Δ*f* corrects for the asymmetric lineshape as described earlier. The red lines (labelled as ‘Model 1’) in Fig. 3 show the results of these fits using standard simulations. We note that the transition to the 2P_e_ state is allowed when the laser polarization is not perfectly perpendicular to the *B* field. This could arise from the slight angle between the laser and the magnetic field (maximum 4° at the edge of our trap) or from a possible nonlinear component in the polarization of the 121-nm light (expected to be of the order of 10% or less). The frequency of the 1S–2P_e_ transition is well separated from that of the 1S–2P_c_ transition (by about −3.5 GHz), and its predicted intensity is very small (less than a few per cent of that for the 1S–2P_c_ transition), hence it was ignored in the analysis.

### Transition-frequency uncertainties

Extensive studies were performed to quantify the uncertainties in our frequency determination. The standard simulated spectra reproduce the observed lineshape reasonably well without any fine-tuning (Extended Data Fig. 1). The sensitivity of the obtained resonance frequency *f*_res_(exp) to the input parameters in the simulation was studied by varying these input parameters and repeating the same analysis.

The standard input to the simulation and the range of the parameters studied (given in parentheses), were as follows. Laser pulse energies, 500 pJ (350 pJ, 800 pJ); laser line linewidth, 65 MHz (50 MHz, 80 MHZ); relative magnitude of the laser sideband (present at +90 MHz with respect to the main band owing to multimode lasing in the 730-nm amplification cavity), 10% (0%, 25%); radial position displacement of the laser beam: 0 mm (0 mm, 3 mm); initial quantum state of antihydrogen at formation: *n* = 30 (1, 30); initial diameter of the cloud of antihydrogen: 0.45 mm (0.45 mm, 0.90 mm); temperature of antihydrogen at formation (before trapping): 15 K (1 K, 15 K).

An alternative fitting method was also used to study the robustness of our procedure. Here, the lineshape function GE was fitted to the data without using constraints from the simulated spectrum. From the fit, *f*_peak_(exp) was extracted for each transition, and the experimental resonance frequency was determined as *f*_res_(exp) = *f*_peak_(exp) − Δ*f*, where the offset Δ*f* from the standard simulation was assumed. The lineshapes given by these fits are shown by blue lines (labelled as ‘Model 2’) in Fig. 3.

The results of the analyses using the simulations with varied input parameters, as well as alternative fitting models, are given by red lines in Extended Data Fig. 1, which illustrates that the dependence on the details of the fitting procedure is small. The variations of the extracted frequency *f*_res_(exp) in these studies (both with different simulation inputs and different fitting methods) were generally within the statistical uncertainties of these fits. We took the largest deviations in *f*_res_(exp) among these studies as a measure of the fitting-model dependence (Table 3).

It should be noted that our evaluation of the fitting-model dependence systematics relies on the GE model being a reasonable representation of the simulated data. This agreement is qualitatively illustrated in Extended Data Fig. 1. Quantitatively, for the simulations with the standard input parameters, the *χ*^2^ per degree of freedom (DOF) ranges from 1.2 to 2.5 (with an average of 1.8), where DOF = 8. When the input parameters are varied in the fits to the data, the *χ*^2^ per DOF ranges from 1.0 to 3.9, with an average of 2.1. The simulation statistics were roughly a factor of 2–4 greater than the data; hence, the uncertainties arising from our analytical model of the simulation lineshape are small.

The sources of uncertainty in the transition frequencies can be summarized as follows (we note that the frequency uncertainties at 730 nm should be multiplied by a factor of 6 to give those at 121 nm): (a) Wavemeter drift: this is due to temperature-induced drift of the wavemeter readings, which was estimated from offline studies to be about 20 MHz K^−1^ at 730 nm. Given the recorded temperature variation of ±0.25 K, we assigned an error of ±5 MHz at 730 nm. We note that a temperature drift during our 2-h measurements would result in a broadening of the observed linewidth. This effect would be also taken into account partly by the fitting-model uncertainty discussed above. Therefore, there is a possibly of partial double counting, but we conservatively list both effects separately. (b) Wavemeter offset: an offset of the He–Ne laser calibration source, estimated to be ±3 MHz at 730 nm by offline calibration. (c) 730-nm cavity resonance-frequency correction: the frequency of the generated 730-nm pulse was measured to be shifted from that of the continuous-wave 730-nm seed laser. This shift of about 10 MHz at 730 nm was regularly monitored, and was corrected for in our frequency determination. We conservatively assign an error of $10/\sqrt{12}=3\,{\rm{MHz}}$ to this correction at 730 nm (the standard deviation of a uniform distribution with a width of 10 MHz). (d) Residual 1S_c_ state contamination: our earlier studies with shorter running times^11,22^ indicate there is a residual population of the order of 5% of the 1S_c_ state after the microwave-driven clearing procedure, which was corrected for in the analysis above. We estimate the error in this correction by analysing the data assuming no residual 1S_c_ population. We take 68% of the differences between the two analysis results (33.5 MHz and 24 MHz for the 2P_c_ and 2P_f_ transitions, respectively) as 1*σ* uncertainties in the correction. (e) Magnetic field: the field at the magnetic minimum of the ALPHA-2 trap was measured in situ using the electron cyclotron resonance (ECR) method^35^. A conservative uncertainty of 10 MHz in the ECR measurement gives a *B* field error of 3.6 × 10^−4^ T, which in turn gives frequency errors of 5 MHz and 8 MHz for the 1S–2P_c_ and 1S–2P_f_ transitions, respectively, at 1 T. We take these values as a measure of the uncertainty due both to the absolute value and to the run-to-run stability of the *B* field. We note that the frequency uncertainty in the 1S–2S transition due to *B*-field variations is negligible for our purposes^11^. (f) Statistical uncertainties of the fit: these represent statistical uncertainties in the fit both from the experimental data and from the simulations. (g) Model uncertainties: described above.

The total errors for each transition are given by the quadratic sum of errors (a)–(g). Care must be taken when taking an average or a difference of the transition frequencies. Here we assume that error (b), the wavemeter offset, introduces a common offset to all the data series. The other errors are assumed to be uncorrelated across the dataset. The resulting combined uncertainty for the transition frequencies of antihydrogen is 39 MHz or 16 ppb (Fig. 4, average value). We expect that virtually all of the uncertainties can be considerably reduced in the near future owing to increased statistics and improved control of the systematics.

### Determination of the fine-structure splitting and the Lamb shift of antihydrogen

To analyse the Zeeman-shifted energy levels of antihydrogen in the 2P state, we used the following Hamiltonian for the 2P state, which includes the field-free Hamiltonian (${\hat{H}}_{0}$), the fine-structure Hamiltonian (${\hat{H}}_{{\rm{fs}}}$), the Zeeman Hamiltonian (${\hat{H}}_{{\rm{Z}}}$) and the hyperfine-structure Hamiltonian (${\hat{H}}_{{\rm{hf}}}$):

1 $$\hat{H}={\hat{H}}_{0}+{\hat{H}}_{{\rm{fs}}}+{\hat{H}}_{{\rm{Z}}}+{\hat{H}}_{{\rm{hf}}}$$

2 $${\hat{H}}_{{\rm{fs}}}=\frac{2}{3}{\bar{ {\mathcal E} }}_{{\rm{fs}}}\left(\frac{1}{{\hbar }^{2}}{{\bf{L}}}_{\bar{e}}\cdot {{\bf{S}}}_{\bar{e}}+1\right)$$

3 $${\hat{H}}_{{\rm{Z}}}=-\frac{2{\mu }_{\bar{e}}({\rm{2P}})}{\hbar }{{\bf{S}}}_{\bar{e}}\cdot {\bf{B}}-\frac{2{\mu }_{\bar{p}}}{\hbar }{{\bf{I}}}_{\bar{p}}\cdot {\bf{B}}+\frac{{\tilde{\mu }}_{\bar{B}}}{\hbar }{{\bf{L}}}_{\bar{e}}\cdot {\bf{B}}$$

4 $${\hat{H}}_{{\rm{hf}}}=\frac{{\bar{C}}_{IL}}{{\hbar }^{2}}{{\bf{I}}}_{\bar{p}}\cdot {{\bf{L}}}_{\bar{e}}+\frac{{\bar{C}}_{IS}}{{\hbar }^{2}}[{{\bf{I}}}_{\bar{p}}\cdot {{\bf{S}}}_{\bar{e}}-3({{\bf{I}}}_{\bar{p}}\cdot {\bf{r}})({{\bf{S}}}_{\bar{e}}\cdot {\bf{r}})]$$

Here, ${{\bf{L}}}_{\bar{e}}$ is the orbital angular momentum of the positron, ${{\bf{S}}}_{\bar{e}}$ is the spin angular momentum of the positron, ${{\bf{I}}}_{\bar{p}}$ is the nuclear spin angular momentum of the antiproton and **r** is the position vector of the positron. ${\bar{ {\mathcal E} }}_{{\rm{fs}}}$ is the fine-structure splitting of antihydrogen at zero field. The magnetic moments of the positron and antiproton are given by ${\mu }_{\bar{e}}(2{\rm{P}})=\frac{|{\bar{g}}_{{\rm{s}}}|}{2}\frac{|\bar{e}|\hbar }{2{m}_{\bar{e}}}\left(1-\frac{{\alpha }^{2}}{10}\right)$ and ${\mu }_{\bar{p}}=-\frac{|{\bar{g}}_{{\rm{p}}}|}{2}\frac{|\bar{e}|\hbar }{2{m}_{\bar{p}}}$ where ${\bar{g}}_{{\rm{s}}}$ and ${\bar{g}}_{{\rm{p}}}$ are the positron spin and antiproton *g*-factors, respectively, $\bar{e}$ and ${m}_{\bar{e}}$ are the charge and mass of the positron, correspondingly, and *α* is the fine-structure constant. The last term of equation (4) is the Zeeman interaction due to the orbital angular momentum of the positron with magnetic moment of ${\tilde{\mu }}_{\bar{B}}=-\left(1-\frac{{m}_{\bar{e}}}{{m}_{\bar{p}}}\right)\frac{|\bar{e}|\hbar }{2{m}_{\bar{e}}}$, where ${m}_{\bar{p}}$ is the mass of the antiproton. ${\bar{C}}_{IL}$ is the hyperfine-coupling constant due to the antiproton spin and the orbital angular momentum of the positron, and ${\bar{C}}_{IS}$ is the hyperfine interaction due to the magnetic dipole–dipole interaction.

For the analysis of the classic Lamb shift (${\bar{ {\mathcal E} }}_{{\rm{Lamb}}}$) and the fine-structure (${\bar{ {\mathcal E} }}_{{\rm{fs}}}$) parameters of antihydrogen, we assumed that the absolute values of the three magnetic moments (${\mu }_{\bar{e}}$, ${\mu }_{\bar{p}}$ and ${\tilde{\mu }}_{\bar{B}}$) are the same as those of hydrogen. Previous measurements of the basic properties of antiparticles are consistent with this assumption. The hyperfine-coupling constants are also assumed to be those of hydrogen^38^, ${\bar{C}}_{IL}=22.2\,{\rm{MHz}}$ and ${\bar{C}}_{IS}=-22.2\,{\rm{MHz}}$.

Our measurements determine the energy levels, with respect to the 1S ground state, of two of the Zeeman sublevels in the *n* = 2 positronic manifold of antihydrogen at a magnetic field of 1.0329 T. Specifically, the 2P_f_ state belongs to the 2P_1/2_ manifold, and the 2P_c_ state belongs to the 2P_3/2_ manifold (see Fig. 1). We combine these results with our previous measurement of the 1S_d_–2S_d_ transition^7^ and assume the validity of the standard Zeeman and hyperfine interactions to derive the fine-structure splitting ${\bar{ {\mathcal E} }}_{{\rm{fs}}}$ (that is, the energy difference between 2P_1/2_ and 2P_3/2_), and the classic Lamb shift ${\bar{ {\mathcal E} }}_{{\rm{Lamb}}}$ (that is, the energy difference between 2S_1/2_ and 2P_1/2_), both defined at zero field.

Taking into account the hyperfine splitting, we find the energy separation between the 2P_c−_ and 2P_f−_ levels at 1.0329 T to be 14.945 ± 0.0975 GHz, from the difference of the weighted average values of the observed transition frequencies. Furthermore, we obtain the separation between the 2S_d_ and 2P_c−_ levels to be Δ*E*(2S, 2P_c_) = 9,832 ± 49 MHz, and that between the 2S_d_ and 2P_f−_ levels to be Δ*E*(2S, 2P_f_) = 24,778 ± 60 MHz, in the same field. The sum and the difference of the two quantities, Δ*E*(2S, 2P_c_) and Δ*E*(2S, 2P_f_), can be expressed by the following equations, which are based on the standard Hamiltonian of the hydrogen atom in a magnetic field *B* (refs. ^36,37^). We neglect terms that contribute less than 1 MHz.

5 $$\begin{array}{c}\Delta E(2{\rm{S}},{2{\rm{P}}}_{{\rm{f}}})-\Delta E(2{\rm{S}},{2{\rm{P}}}_{{\rm{c}}})=\\ 2{E}_{1}(-B)+\frac{1}{2}{\bar{C}}_{IL}\cos (2\sigma )+\frac{1}{10}[\cos (2\sigma )-3\sqrt{2}\sin (2\sigma )]{\bar{C}}_{IS}\end{array}$$

6 $$\begin{array}{c}\Delta E(2{\rm{S}},{2{\rm{P}}}_{{\rm{f}}})+\Delta E(2{\rm{S}},{2{\rm{P}}}_{{\rm{c}}})=\\ 2{\bar{{\mathcal{E}}}}_{{\rm{L}}{\rm{a}}{\rm{m}}{\rm{b}}}-2{\bar{{\mathcal{E}}}}_{{\rm{f}}{\rm{s}}}+\frac{1}{2}{\bar{C}}_{IL}-\frac{3}{10}{\bar{C}}_{IS}+\frac{1}{2}{\bar{{\mathcal{E}}}}_{{\rm{h}}{\rm{f}}}(2{\rm{S}})-[2{\mu }_{\bar{e}}(2{\rm{P}})+{\mathop{\mu }\limits^{ \sim }}_{\bar{B}}]B\end{array}$$

where

$${E}_{1}(-B)=\sqrt{{\left\{\frac{1}{6}{\bar{ {\mathcal E} }}_{{\rm{fs}}}-B\left[{\mu }_{\bar{e}}({\rm{2P}})+\frac{1}{2}{\tilde{\mu }}_{\bar{B}}\right]\right\}}^{2}+\frac{2}{9}{\bar{ {\mathcal E} }}_{{\rm{fs}}}^{2}}$$

and

$$\tan \,\sigma =\frac{-{\bar{{\mathcal{E}}}}_{{\rm{f}}{\rm{s}}}+[6{\mu }_{\bar{e}}(2{\rm{P}})+3{\mathop{\mu }\limits^{ \sim }}_{\bar{B}}]B+6{E}_{1}(-B)}{2\sqrt{2}{\bar{{\mathcal{E}}}}_{{\rm{f}}{\rm{s}}}}$$

Here, ${\bar{ {\mathcal E} }}_{{\rm{hf}}}(2{\rm{S}})$ is the hyperfine splitting in the 2S state at zero field.

Finally, using the CODATA 2014 values of the fundamental constants for the hydrogen atom^39^, the fine-structure splitting ${\bar{ {\mathcal E} }}_{{\rm{hf}}}$ and the classic Lamb shift ${\bar{ {\mathcal E} }}_{{\rm{Lamb}}}$ of the antihydrogen atom are determined by numerically solving equations (5) and (6) with the measured energy-level differences given in Table 2 as input.

### Hydrogen transition frequencies in a magnetic field

From zero-field measurements in hydrogen for the 1S_1/2_–2S_1/2_ (ref. ^40^), 2S_1/2_–2P_1/2_ (ref. ^41^) and 2P_1/2_–2P_3/2_ (ref. ^42^) transitions, we obtain hyperfine centroid frequencies of

$$\begin{array}{c}{\mathrm{1S}\mbox{--}\mathrm{2P}}_{1/2}\mathrm{\ transition:\; 2,466,060,355\; MHz}\\ {\mathrm{1S}\mbox{--}\mathrm{2P}}_{3/2}\mathrm{\ transition:\; 2,466,071,324\; MHz}\end{array}$$

The transition frequencies at 1.0329 T (Table 2) are calculated by evaluating corrections assuming the standard Zeeman, fine-structure and hyperfine interactions in a magnetic field^36,37^ and using the current CODATA values of the fundamental constants^39^. The precision of our calculations is better than 1 MHz.

In comparing the hydrogen values with the measured antihydrogen frequencies in Table 2 and Fig. 4, the value of the magnetic field was assumed to be exact for the hydrogen case.

## Online content

Any methods, additional references, Nature Research reporting summaries, source data, extended data, supplementary information, acknowledgements, peer review information; details of author contributions and competing interests; and statements of data and code availability are available at 10.1038/s41586-020-2006-5.

## Data Availability

The datasets generated and/or analysed during the current study are available from J.S.H. on reasonable request.
