# Author Correction: Investigation of the fine structure of antihydrogen

**DOI:** 10.1038/s41586-021-03367-9

**Published:** 2021-05-18

**Authors:** The ALPHA Collaboration, M. Ahmadi, B. X. R. Alves, C. J. Baker, W. Bertsche, A. Capra, C. Carruth, C. L. Cesar, M. Charlton, S. Cohen, R. Collister, S. Eriksson, A. Evans, N. Evetts, J. Fajans, T. Friesen, M. C. Fujiwara, D. R. Gill, P. Granum, J. S. Hangst, W. N. Hardy, M. E. Hayden, E. D. Hunter, C. A. Isaac, M. A. Johnson, J. M. Jones, S. A. Jones, S. Jonsell, A. Khramov, P. Knapp, L. Kurchaninov, N. Madsen, D. Maxwell, J. T. K. McKenna, S. Menary, J. M. Michan, T. Momose, J. J. Munich, K. Olchanski, A. Olin, P. Pusa, C. Ø. Rasmussen, F. Robicheaux, R. L. Sacramento, M. Sameed, E. Sarid, D. M. Silveira, C. So, D. M. Starko, G. Stutter, T. D. Tharp, R. I. Thompson, D. P. van der Werf, J. S. Wurtele

**Affiliations:** 1 Department of Physics, University of Liverpool, Liverpool, UK; 2 Department of Physics and Astronomy, Aarhus University, Aarhus, Denmark; 3 Department of Physics, College of Science, Swansea University, Swansea, UK; 4 School of Physics and Astronomy, University of Manchester, Manchester, UK; 5 Cockcroft Institute, Warrington, UK; 6 TRIUMF, Vancouver, British Columbia Canada; 7 Department of Physics, University of California at Berkeley, Berkeley, CA USA; 8 Instituto de Fisica, Universidade Federal do Rio de Janeiro, Rio de Janeiro, Brazil; 9 Department of Physics, Ben-Gurion University of the Negev, Beer-Sheva, Israel; 10 Department of Physics and Astronomy, University of Calgary, Calgary, Alberta Canada; 11 Department of Physics and Astronomy, University of British Columbia, Vancouver, British Columbia Canada; 12 Department of Physics, Simon Fraser University, Burnaby, British Columbia Canada; 13 Department of Physics, Stockholm University, Stockholm, Sweden; 14 Department of Physics and Astronomy, York University, Toronto, Ontario Canada; 15 École Polytechnique Fédérale de Lausanne (EPFL), Swiss Plasma Center (SPC), Lausanne, Switzerland; 16 Department of Chemistry, University of British Columbia, Vancouver, British Columbia Canada; 17 Department of Physics and Astronomy, University of Victoria, Victoria, British Columbia Canada; 18 Department of Physics and Astronomy, Purdue University, West Lafayette, IN USA; 19 Soreq NRC, Yavne, Israel; 20 Physics Department, Marquette University, Milwaukee, WI USA; 21 IRFU, CEA/Saclay, Gif-sur-Yvette, France

**Keywords:** Exotic atoms and molecules, Experimental particle physics

## Abstract

A Correction to this paper has been published: https://doi.org/10.1038/s41586-021-03367-9.

Correction to: *Nature* 10.1038/s41586-020-2006-5Published online 19 February 2020

In this Article, in Fig. 1 and its legend, the 1S_b_ state should read |↑⇑⟩ (not |↑⇓⟩) and the 1S_a_ state should read |↑⇓⟩ (not |↑⇑⟩). Also in the legend to Fig. 1, the sentence “The symbol (↓,↑) in the figure indicates that the positron spin states are mixed for the 2P_c_ and 2P_f_ states” should read “The symbol (0, ↑) or (1, ↓) for the 2P states in the figure indicates that the positron spin states are mixed for the 2P_c_ and 2P_f_ states, where the first value (0 or 1) in the label corresponds to the magnetic quantum number *m*_*L*_ of the orbital angular momentum *L* = 1 in the 2P state, while the second label ↑ or ↓ indicates the positron spin.” In Fig. 3b, the label ‘Singly spin-polarized’ should read ‘Doubly spin-polarized’. These errors do not affect any results or conclusions. The original Article has been corrected online.

